# Supplementary material for: Blood draw site and analytic device influence hemoglobin measurements
Source: PLoS One. 2022 Nov 30;17(11):e0278350. doi: 10.1371/journal.pone.0278350 (PMC9710840; doi:10.1371/journal.pone.0278350)

Supplemental Figure 1: Summary of demographic information on study participants. Pie chart of gender, age-range, race/ethnicity, and body mass index (BMI) for participants. Gender, age, and ethnicity were provided directly by participants. BMI was calculated from height and weight measured on each participant.

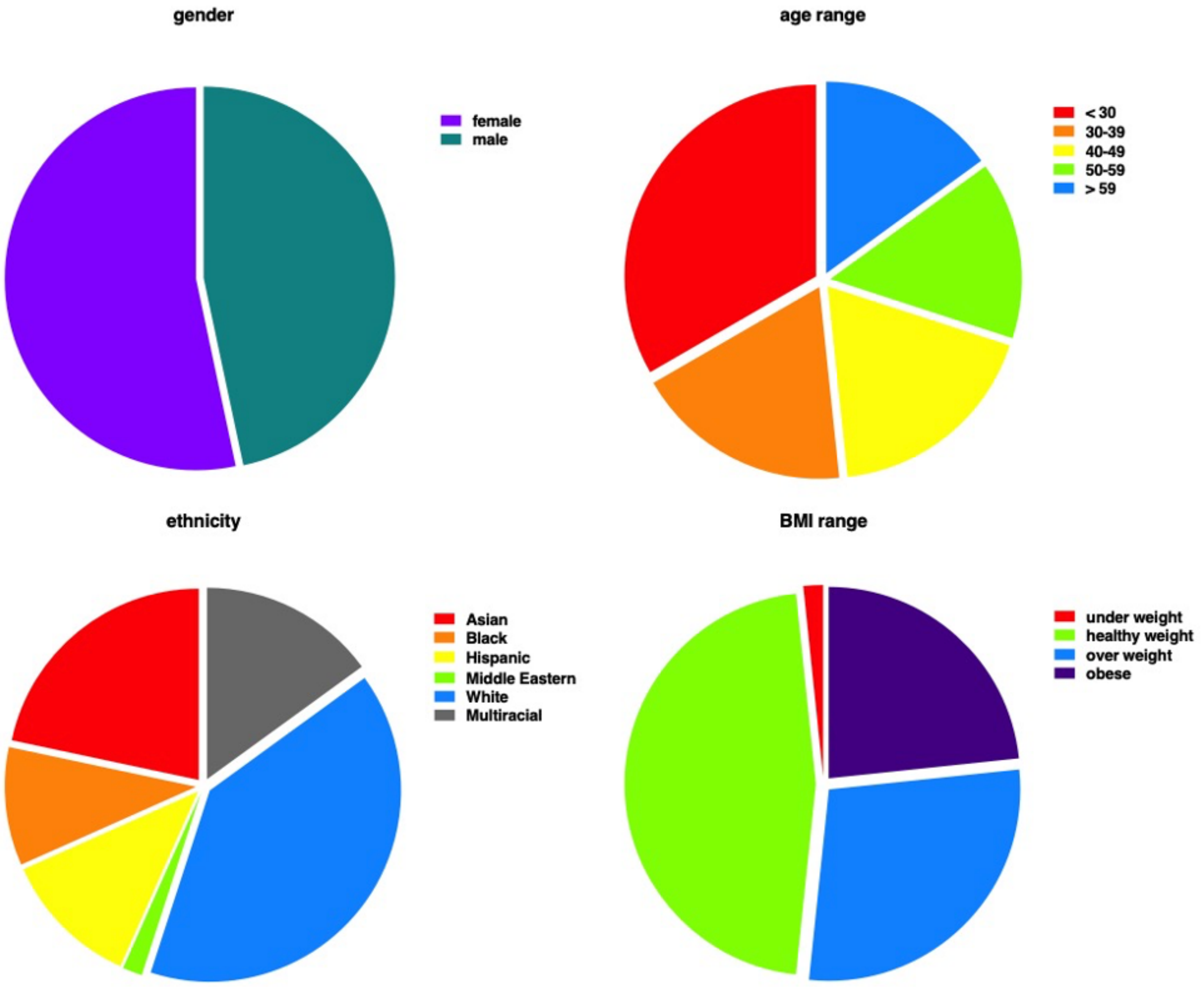

Supplement: S1 Fig — Pie chart of gender, age-range, race/ethnicity, and body mass index (BMI) for participants. Gender, age, and ethnicity were provided directly by participants. BMI was calculated from height and weight measured on each participant. (PDF) [file pone.0278350.s001.pdf]
