# Supplementary material for: Blood draw site and analytic device influence hemoglobin measurements
Source: PLoS One. 2022 Nov 30;17(11):e0278350. doi: 10.1371/journal.pone.0278350 (PMC9710840; doi:10.1371/journal.pone.0278350)

Supplemental Figure 2: Hemoglobin concentration is not different in blood collected with blood collection tubes (BCTs) from different manufacturers. Correlation and Bland-Altman plots for Hb values determined in venous blood collected in BCTs made by BD or Sarstedt using (A) ADVIA-2120, (B) HemoCue 201+, and (C) HemoCue 301. The correlation plots show the capillary and venous Hb values (mean ± SEM) proximity to line of concordance (solid black line), as well as linear regression (solid red line) ± 95% confidence interval (red dotted line) for the group. Each circle represents a single participant, with participant ID number to the immediate right. The Bland-Altman plot shows a bias of (A) 0.127 ± 0.469 g/dL for Sarstedt over venous BD for ADVIA-2120, but a bias of (B) 0.491 ± 1.033 g/dL and (C) 0.042 ± 0.368 g/dL for HemoCue 201+ and HemoCue 301, respectively.

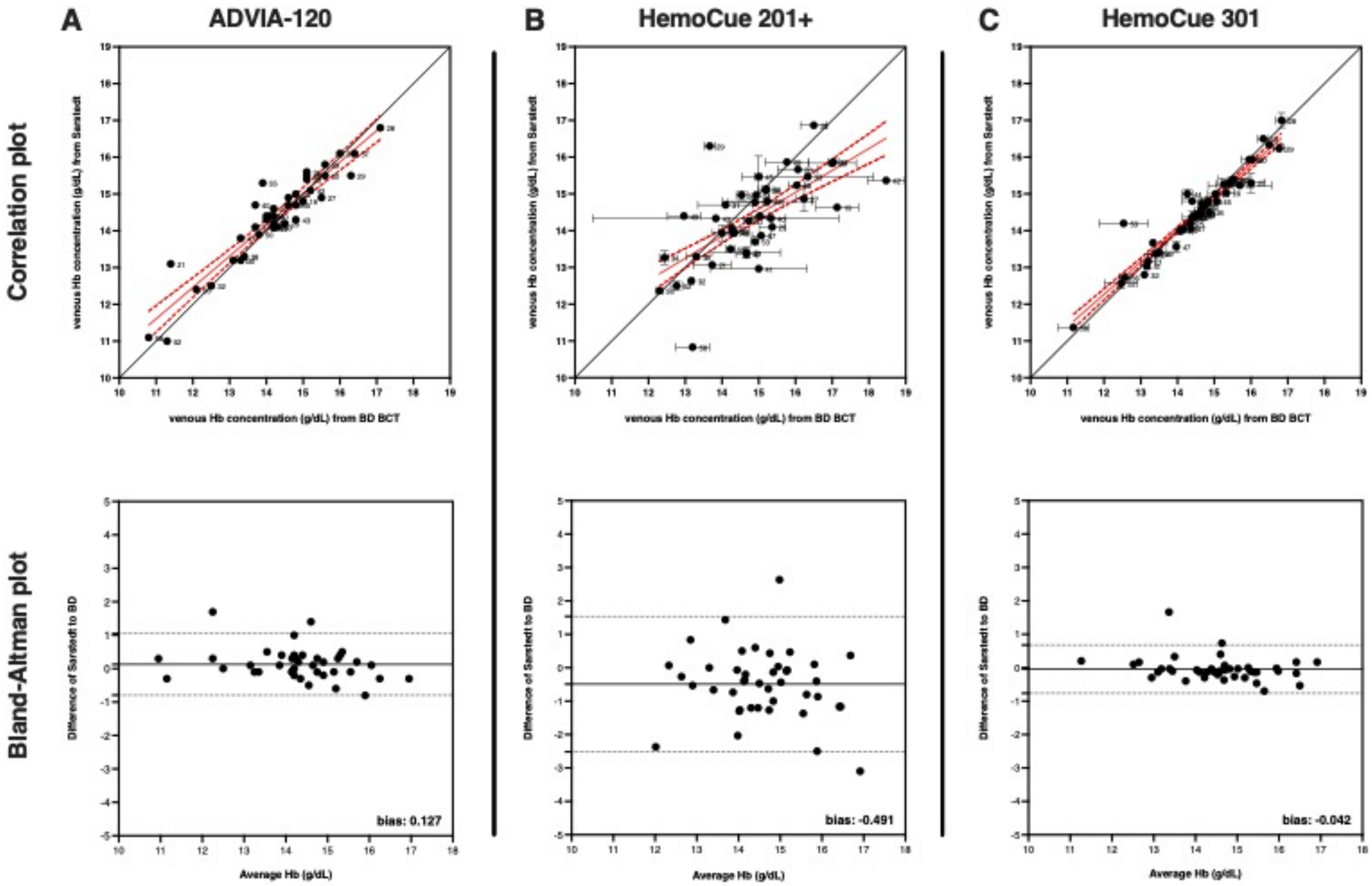

Supplement: S2 Fig — Correlation and Bland-Altman plots for Hb values determined in venous blood collected in BCTs made by BD or Sarstedt using (A) ADVIA-2120, (B) HemoCue 201+, and (C) HemoCue 301. The correlation plots show the capillary and venous Hb values (mean ± SEM) proximity to line of concordance (solid black line), as well as linear regression (solid red line) ± 95% confidence interval (red dotted line) for the group. Each circle represents a single participant, with participant ID number to the immediate right. The Bland-Altman plot shows a bias of (A) 0.127 ± 0.469 g/dL for Sarstedt over venous BD for ADVIA-2120, but a bias of (B) 0.491 ± 1.033 g/dL and (C) 0.042 ± 0.368 g/dL for HemoCue 201+ and HemoCue 301, respectively. (PDF) [file pone.0278350.s002.pdf]
