# Supplementary material for: Blood draw site and analytic device influence hemoglobin measurements
Source: PLoS One. 2022 Nov 30;17(11):e0278350. doi: 10.1371/journal.pone.0278350 (PMC9710840; doi:10.1371/journal.pone.0278350)
Supplement: S1 Table — Step-by-step procedure for sequential collection of venous and capillary blood samples for this study are listed. “BCT” indicates blood collection tubes. (PDF) [file pone.0278350.s003.pdf]

Supplemental Table 1: Procedure for venous and capillary blood draw. Step-by-step procedure for sequential collection of venous and capillary blood samples for this study are listed. “BCT” indicates blood collection tubes.

Finger-prick procedures:

1. Identify the non-dominant hand of participant for fingerstick.
2. Warm selected hand in warm water for 5-10 minutes.
3. Organize the blood collection tubes, lancets, and other supplies.
4. Have participants stand leaning against a bench for support and let arms fall naturally.
5. Clean thumb with alcohol disinfectant wipe and allow to air dry.
6. Place lancet on side of distal phalanx of thumb and press the trigger.
7. Wipe away first drop of blood with clean sterile pad.
8. Place an open capillary BCT to thumb and allow blood to drop and collect into BCT.
9. Do not squeeze or milk finger to avoid hemolysis and addition of interstitial fluid.
10. Once blood volume in BCT approaches 500µl, close BCT and invert 4-6 times.
11. If thumb continues to provide blood, use new capillary BCT to collect, up to total of 4.
12. If first capillary volume was <1ml, repeats steps 5-12 with 4th finger.
13. Hold BCTs at room temp and transfer to laboratory in <60 min.

Venipuncture procedures: Participants told to hydrate 1 hour before clinical visit.

1. Identify the non-dominant arm of participant for venipuncture.
2. Organize the blood collection tubes, syringe, and other supplies.
3. Have participants sit in blood draw chair and place are on phlebotomy platform.
4. Identify optimal site for venipuncture, usually the antecubital vein.
5. Clean site with alcohol disinfectant wipe and allow to air dry.
6. Proceed with phlebotomy using standard protocols using conventional tube order procedures.
7. Once blood volume in BCT approaches 6ml (BD) or 7.5ml (Sarstedt), withdraw syringe and invert 4-6 times.
8. Continue to collect venous blood until desired number of BCTs is complete.
9. Hold BCTs at room temp and transfer to laboratory in <60 min.
